# Drug-Induced Phase Separation in Polyelectrolyte Microgels

**DOI:** 10.3390/gels8010004

**Published:** 2021-12-22

**Authors:** Yassir Al-Tikriti, Per Hansson

**Affiliations:** 1 Department of Pharmacy, Uppsala University, P.O. Box 580, 75123 Uppsala, Sweden; yassir.al-tikriti@ilk.uu.se; 2 Department of Medicinal Chemistry, Uppsala University, P.O. Box 574, 75123 Uppsala, Sweden

**Keywords:** microgel, drug, amphiphile, phase transition, phase separation, microscopy, micropipette, binding isotherm, swelling

## Abstract

Polyelectrolyte microgels may undergo volume phase transition upon loading and the release of amphiphilic molecules, a process important in drug delivery. The new phase is “born” in the outermost gel layers, whereby it grows inward as a shell with a sharp boundary to the “mother” phase (core). The swelling and collapse transitions have previously been studied with microgels in large solution volumes, where they go to completion. Our hypothesis is that the boundary between core and shell is stabilized by thermodynamic factors, and thus that collapsed and swollen phases should be able to also coexist at equilibrium. We investigated the interaction between sodium polyacrylate (PA) microgel networks (diameter: 400–850 µm) and the amphiphilic drug amitriptyline hydrochloride (AMT) in the presence of NaCl/phosphate buffer of ionic strength (I) 10 and 155 mM. We used a specially constructed microscopy cell and micromanipulators to study the size and internal morphology of single microgels equilibrated in small liquid volumes of AMT solution. To probe the distribution of AMT micelles we used the fluorescent probe rhodamine B. The amount of AMT in the microgel was determined by a spectrophotometric technique. In separate experiments we studied the binding of AMT and the distribution between different microgels in a suspension. We found that collapsed, AMT-rich, and swollen AMT-lean phases coexisted in equilibrium or as long-lived metastable states at intermediate drug loading levels. In single microgels at I = 10 mM, the collapsed phase formed after loading deviated from the core-shell configuration by forming either discrete domains near the gel boundary or a calotte shaped domain. At I = 155 mM, single microgels, initially fully collapsed, displayed a swollen shell and a collapsed core after partial release of the AMT load. Suspensions displayed a bimodal distribution of swollen and collapsed microgels. The results support the hypothesis that the boundary between collapsed and swollen phases in the same microgel is stabilized by thermodynamic factors.

## 1. Introduction

Polyelectrolyte hydrogels are soft and elastic materials, consisting of slightly cross-linked charged polymers with interesting chemical and mechanical properties [1]. They have the capacity to absorb and retain large amounts of water per gram of dry material even under load. This makes them useful as super absorbents and filler materials, e.g., in cosmetic surgery [2]. The high water content provides them with bioadhesive properties and the capacity to host water soluble species, explaining why they are used as cell supporting scaffoldings in tissue engineering [3] and as sustained release depots in pharmaceutical applications [4,5,6,7,8,9,10]. Charged gel networks attract ionic species of opposite charge and have therefore been used for decades in ion exchange resins [11] and protein purification systems. For the same reason they are also interesting as storage and release systems for ionic drugs [3,4,5,6,7,8,9,12,13,14]. In many applications the polyelectrolyte networks are spherical microgel particles with diameters in the range 10–1000 µm. There are a number of research papers showing that proteins and peptides can be loaded onto such microgels at low ionic strength and subsequently released at elevated ionic strengths [4,14,15,16,17,18,19,20,21,22,23,24,25,26,27,28,29,30,31,32,33]. The electrostatic interaction with the charged network does not seem to denature the molecules; instead the environment inside the microgel has been shown to protect them against degrading species [34,35]. The reversible loading-release property is particularly efficient for multivalent proteins and species forming aggregates of multivalent charge, such as micelle forming surfactants [28,31,36,37,38,39,40,41,42,43,44,45]. At low ionic strength the gain in entropy from replacing a large number of network counterions with a small number of multivalent species provides a strong driving force for binding. However, the simultaneous lowering of the osmotic swelling pressure leads to deswelling of the microgels [46]. For species of high charge density, cohesive electrostatic forces may give rise to a volume phase transition (VPT), whereby massive binding takes place at a critical concentration in the solution, typically transforming the microgels into dense complex phases of volume about two orders of magnitude lower than in the swollen state prior to binding [22,37,39,40,47]. During the reverse process, when fully loaded microgels release their cargo in a physiological environment, the microgels swell but the volume change is smaller than during loading because of the higher ionic strength of the medium (ca. 0.15 M) [36,48]. Interestingly, the swelling can be utilized to improve the performance of microgel delivery systems. A good example is DC bead^®^, a delivery system registered in the market for the treatment of liver cancer [49,50,51,52]. After the loading of doxorubicin, a chemotherapy drug with self-assembling properties, a catheter is used to deposit the microgels locally in the blood vessels in the malignant liver tissue. The release process is followed by swelling of the microgels, which causes embolization, stopping the blood flow to the cancer cells. This is beneficial in reducing the nutrient flow to the malignant cells, concentrating doxorubicin locally, and lowering systemic spreading of the drug, thereby reducing detrimental side effects. In vitro studies show that DC bead microgels have a core/shell internal structure during release of doxorubicin [36,51]. The core is rich in drug molecules and the shell is depleted of drug molecules. Ahnfelt et al. [36] showed that a series self-assembling cationic drug molecules behaved in the same way, and concluded that the core contained aggregates of drug molecules stabilized by hydrophobic interactions, similar to micelles formed by conventional surfactants [53]. They also found that the release rate increased with increasing value of the critical micelle concentration (CMC) of the drug, and proposed a mechanism where the overall rate determining step is the diffusion of single drug molecules through the shell network. Under physiological conditions, the driving force for the release is mainly entropic, since the body acts as a sink. However, according to the proposed mechanism, salt present at the release site affects the release rate by weakening the electrostatic interaction between the drug aggregates and the microgel network, thereby increasing the concentration of free drug monomers in local equilibrium with the micelles at the core/shell boundary. Clearly, to arrive at a quantitative predictive model of the drug release it is necessary to be able to calculate the time evolution of the concentration profile in the depletion layer (shell). So far there are only approximate models available. Ahnfelt et al. used a simple model to describe the local equilibrium between (stationary) micelles and free drug monomers in combination with a transport model where the electrostatic coupling between the diffusion ions was described by means of the Nernst-Planck equation. The model accounted for the development of the depletion layer and produced drug release rates of the correct order of magnitude. To be able to solve the differential equations they assumed a fixed gel volume. This was partly justified by the rather small volume change of DC bead observed experimentally. However, the approximation should be poor for microgels with larger collapse-swelling amplitude. Weakly cross-linked sodium polyacrylate (PA) microgels are more challenging in this respect since they are very responsive. In a previous paper we investigated their interaction with the amphiphilic cationic drug amitriptyline hydrochloride (AMT) from both equilibrium and kinetic perspectives [48]. We found that PA microgels suspended in AMT solutions of ionic strength 10 mM were either swollen or collapsed, with no intermediate semi-swollen states present at equilibrium. We also found core-shell phase separated states in single *macrogels* (~1 mL) in AMT solutions of limited volume. For single microgels in bulk solution of AMT, we observed core-shell phase separation during AMT loading as the microgels underwent VPT from a swollen to collapsed state, but we had no reliable method available to investigate single microgels in equilibrium in a limited volume of AMT solution. A method previously used for ionic surfactants in which the microgels were positioned in water droplets surrounded by oil [31] could not be used since AMT partitioned into the oil phase in the form the non-ionic base. In the present work, we show that the experiment can be performed in sealed glass microtubes, and use the set up to determine at what loading levels swollen and collapsed phases coexist in single microgels, and how the phases are distributed. We use weakly cross-linked PA networks with large mesh size, shown earlier not to prevent amphiphiles from redistributing inside the gels [42,48].

In the previous paper we also showed that the release of AMT at physiological ionic strength was followed by substantial microgel swelling, and that the internal structure of the microgel was of the swollen shell/collapsed core type. We were able to theoretically model the time evolution of the microgel volume during the process by taking into account the swelling of the shell. The rate of AMT mass transport through the shell was shown to be rate determining for the release of the drug as well as swelling of the microgel. The model assumed that local phase equilibrium was maintained at the core/shell boundary. In the present work we test the relevance of that assumption by using our new set up to investigate the thermodynamic stability of the phase boundary in microgels that have released fractions of pre-loaded AMT. To further explore the phase behavior we also investigate microgels suspended in AMT solutions containing 155 mM NaCl and phosphate buffer with respect to binding and distribution of collapsed and swollen phases.

## 2. Results

### 2.1. Drug Loading at 10 mM Ionic Strength

**Figure 1:**
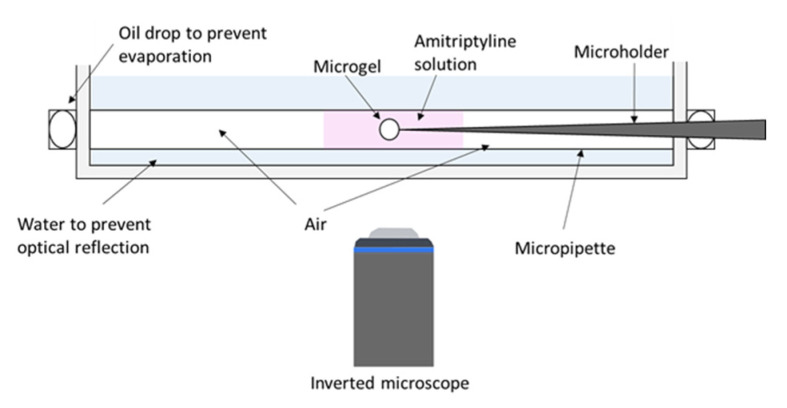
Schematic illustration of the limited-volume experiment set-up. A single microgel is positioned inside AMT solution by a micropipette. The microtube is sealed to prevent evaporation.

A specially designed small-volume microscopy cell was used to investigate the equilibrium states of PA microgels in solutions containing limited amounts of AMT (Figure 1). In each experiment, one single microgel was positioned in a limited volume of AMT solution at ionic strength of 10 mM. With this setup, we were able to load the gel with AMT and capture it at intermediate loading levels. The loading level is described by the binding ratio β, defined in the following way:

(1) $$\beta =\frac{n_{AMT}^{gel}}{n_{PA}^{gel}}$$

where $n_{AMT}^{gel}$ is the number of moles of AMT in the microgel and $n_{PA}^{gel}$ is the number of moles of acrylate segments incorporated in the microgel network, determine as described in Section 5.3. The microgel volume is described by the volume ratio V/$V_{0}$, where V is the actual volume and $V_{0}$ is the volume of the same microgel in an AMT-free solution at ionic strength of 10 mM.

**Figure 2:**
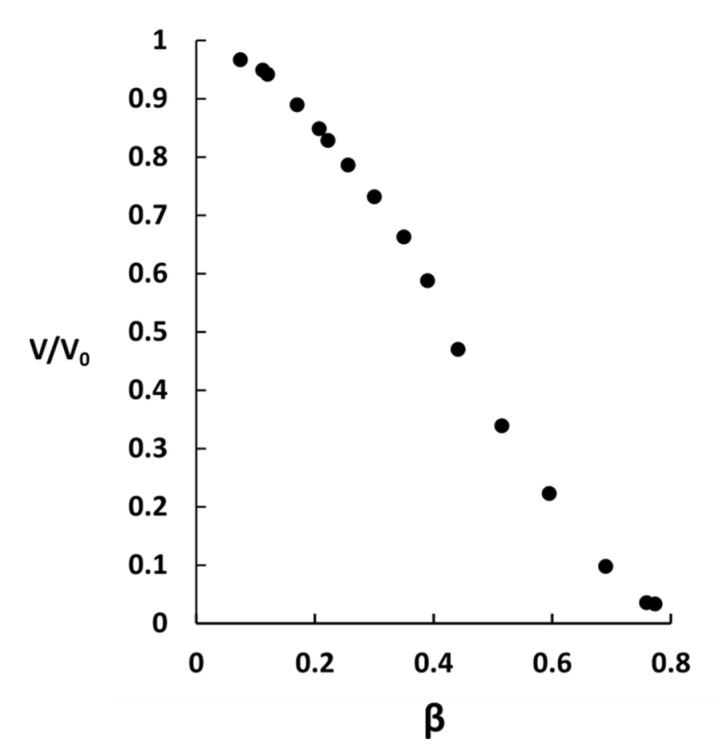
Swelling isotherms for polyacrylate microgels in a limited volume of AMT solution of 10 mM ionic strength. Microgel volume ratio is plotted vs. binding ratio and each point represents a separate experiment.

**Figure 3:**
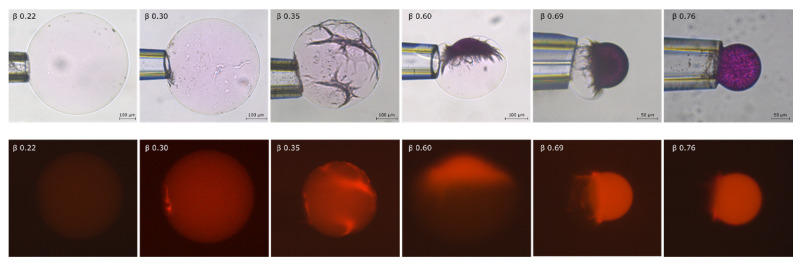
Light microscopy images (**upper row**) and fluorescence images (**lower row**) of polyacrylate microgels after equilibration in a limited volume of AMT solution. Each binding ratio represents a separate experiment.

The volume ratio vs. binding ratio is shown in Figure 2 and microscopy images of microgels from selected points on the curve are shown in Figure 3.

In the lower binding range (β < 0.26), the microgel decreased in size with increasing β but remained homogenous with no sign of phase separation. The decrease was much larger than expected for the addition of the same amount of a simple salt to the solution [48,54], thus indicating that AMT formed micelles inside the microgels. This was confirmed by fluorescence microscopy experiments with small amounts of the hydrophobic fluorescent probe rhodamine B added. The microscopy images show that the fluorescence intensity increased with increasing β and that the probe was homogeneously distributed inside the microgels. As a control experiment, we equilibrated a microgel in a solution of only rhodamine B overnight. There was no change in the size of the microgel and no coloration inside. This shows that the probe only accumulated in the microgels in the presence of AMT micelles.

**Figure 4:**
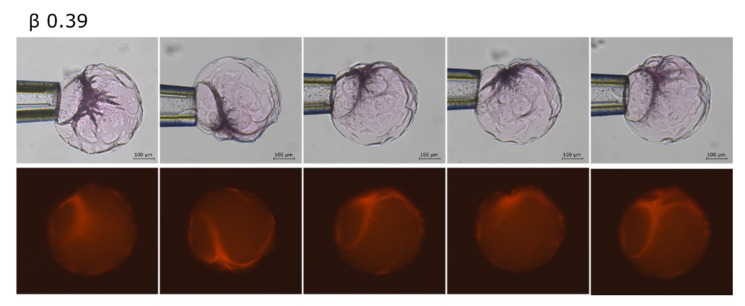
AMT distribution in a polyacrylate microgel after equilibration at binding ratio β = 0.39. Images taken at different angles by rotating the gel holder.

In the binding range 0.26 < β < 0.52 we observed patterns on the microgel surface indicating that collapsed domains co-existed with the swollen core. The size of the collapsed domains increased with increasing β. In this range the volume decreased faster as a function of β than for the lower binding range. The fluorescence images in Figure 3 show that AMT micelles were enriched in the collapsed domains. Figure 4 shows fluorescence images of a microgel with β = 0.39. The gel holder was rotated to expose the microgel from different angles. Rhodamine B was present also in the swollen part after phase separation, suggesting that the swollen part was not entirely free from AMT micelles.

In the binding range 0.52 < β < 0.76, the collapsed AMT rich parts formed a single calotte-shaped domain. The collapsed part increased in size at the expense of the swollen part with increasing β, and the rate of volume change as a function of β was somewhat smaller than in the previous range.

For β > 0.76 the entire microgel was converted to a collapsed AMT rich phase of spherical shape.

**Figure 5:**
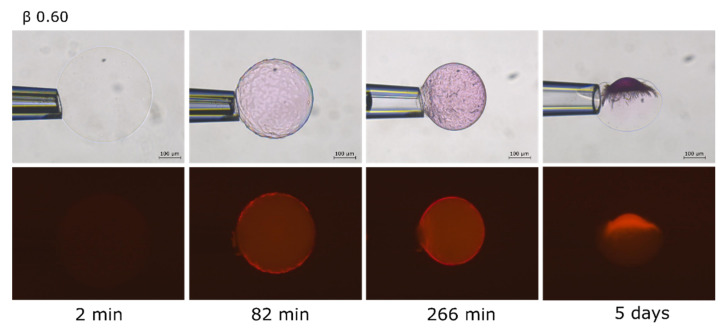
AMT distribution in a polyacrylate microgel at β = 0.60. Images of the microgel taken at different times to show the redistribution of the AMT inside the gel.

Figure 5 shows the evolution of a microgel at different incubation times as it approached the final state with β = 0.60. There was a lag time before the surface pattern appeared. This can be explained by the fact that the gel was placed in a non-stirred liquid, and corresponded to the time needed for the AMT molecules to diffuse from the bulk to the microgel to reach the critical concentration inside needed to nucleate the collapsed phase. After a few hours, a thin AMT rich shell had formed outside the swollen core. However, after five days, AMT had redistributed to a calotte shaped domain coexisting with a deformed swollen phase.

To summarize, the results show that AMT can form micelles uniformly distributed in the microgels. However, above a certain loading level the micelles end up in a collapsed phase. At intermediate loading levels, and after extended incubation periods, the collapsed phase takes on the shape of a calotte, coexisting with a deformed swollen phase.

### 2.2. Phase Coexistence after Partial Release of AMT at Physiological Ionic Strength

**Figure 6:**
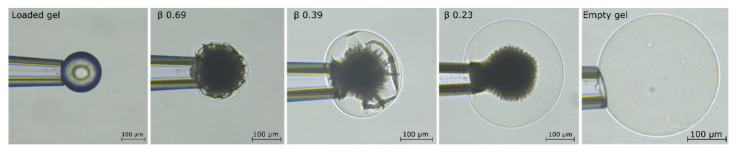
AMT distribution in polyacrylate microgels at different binding ratios in a solution of 155 mM ionic strength. Loaded gel represents overnight equilibration of the gel in 0.7 mM AMT solution of 10 mM ionic strength. Empty gel represents the gel in 155 mM phosphate buffer solution after AMT releases.

To investigate whether two phases can coexist in the same microgel at physiological ionic strength in the solution, we used the small-volume cell in a slightly different way. Single microgels preloaded with AMT (β = 0.85) at low ionic strength (10 mM) were placed inside the cell in a dilute AMT solution of ionic strength 155 mM (150 mM NaCl + 5 mM sodium phosphate buffer). During the incubation time (5 h) the microgel released part of its AMT cargo. The amount released was controlled by the initial amount of AMT in the solution. The amount remaining in the microgel was determined as described in Section 5.4. Figure 6 shows microscopy images of microgels at the end of the incubation period, with β values as indicated. Please note that the circular pattern on the fully loaded microgel is not an inner phase boundary. It is an optical artifact on the curved microgel surface, typical of homogeneous spheres with sufficiently large difference in refractive index from the surrounding medium [55] (e.g., glass beads in air). Clearly, at intermediate binding ratios, a swollen shell developed that increased in thickness with decreasing β. The buckled thin shells at high β show that the network was not fully relaxed after 5 h. Strained regions were observed also at the core boundary at low β. Our interpretation is that at the end of the incubation period the microgels were close to equilibrium with respect to the partitioning of AMT in the polymer network between the swollen and collapsed domains, but not with respect to the elastic deformation energy. Based on that, we conclude that the microgels underwent a gradual phase transformation and that the boundary between the phases should be considered as thermodynamically rather than kinetically stabilized. Still, the arrangement of the swollen and the collapsed phases may not have represented the global equilibrium state of the system, as will be further discussed below.

**Figure 7:**
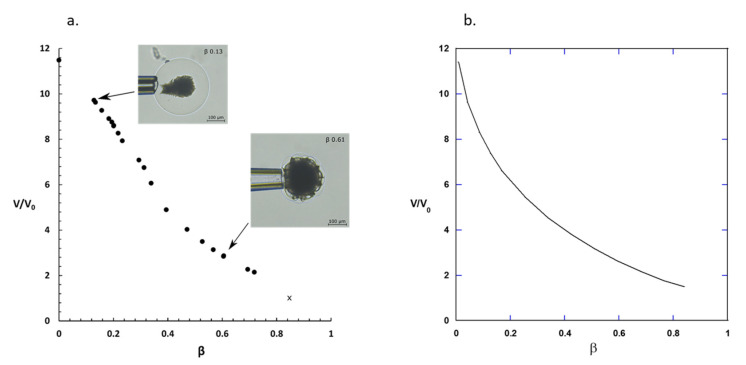
(**a**) Swelling isotherm for polyacrylate microgels in a limited volume of AMT solution of 155 mM ionic strength. Volume ratio is plotted vs. binding ratio and each point represents a separate experiment. The data point (cross) at β = 0.85 is for a homogeneous microgel at 10 mM ionic strength (reference state, *V*/*V*_0_ = 1). (**b**) Theoretical swelling isotherm calculated by means of the model by Al-Tikriti and Hansson [48].

The microgel volume increased with decreasing binding ratio as shown in Figure 7a, where *V*_0_ is the volume of the microgel preloaded with AMT (β = 0.85) at low ionic strength (10 mM). Each data point derives from a separate experiment with an individual microgel as described in Section 5.4. The curve can be divided into two parts. Going from high to low β, the volume increased moderately for β values down to ca. 0.4 where the shell was thin and the core occupied a substantial part of the volume (cf. Figure 6). For β lower than 0.4 the volume increased faster with decreasing β. For a phase conversion without elastic interactions between the phases, one would expect a linear relationship between the gel volume and β. Non-linear swelling isotherms have been reported earlier for phase separated gels with surfactant collapsed shells in solutions of low ionic strength [31,37,42,56]. The swelling isotherm in Figure 7a is the first evidence of the corresponding behavior of phase separated gels with collapsed core and swollen shell.

**Figure 8:**
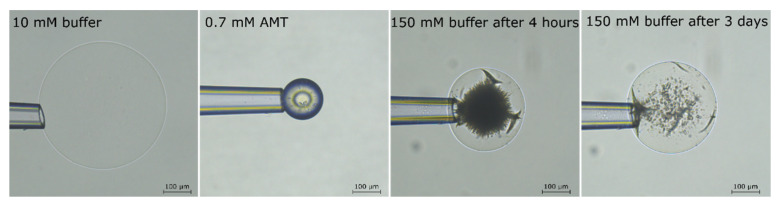
Light microscopy images of a polyacrylate microgel in (from left to right): 10 mM buffer; 0.7 mM AMT solution of 10 mM ionic strength overnight; small volume of AMT solution at 155 mM ionic strength after 4 h and three days, respectively. All pictures show the same microgel particle.

To investigate long term effects for microgels with β in the lower range, we incubated a fully loaded microgel in a limited volume of AMT solution at ionic strength 155 mM for three days. Figure 8 shows pictures of the microgel before and after loading in 0.7 mM AMT solution at 10 mM ionic strength, and after incubation for four hours and three days, respectively, at 155 mM ionic strength. After four hours, a swollen shell had formed with a sharp boundary to the collapsed core. However, after three days, much of the core had dissolved but separate domains of the collapsed phase remained dispersed in the swollen gel matrix. We were not able to measure the amount of AMT bound to the gel in the final state, but the binding ratio after 4 h equilibration could be determined to ca. 0.32 from interpolation of data from the other experiments.

Taken together, the results show that microgels that have attained equilibrium with the solution after having released part of their load remain in a core-shell state for several hours. However, incubation for several days may lead to disintegration and redistribution of the dense phase to other positions in the microgel. The significance of that will be further discussed below.

### 2.3. Phase Coexistence in Microgel Suspension at Elevated Ionic Strength

**Figure 9:**
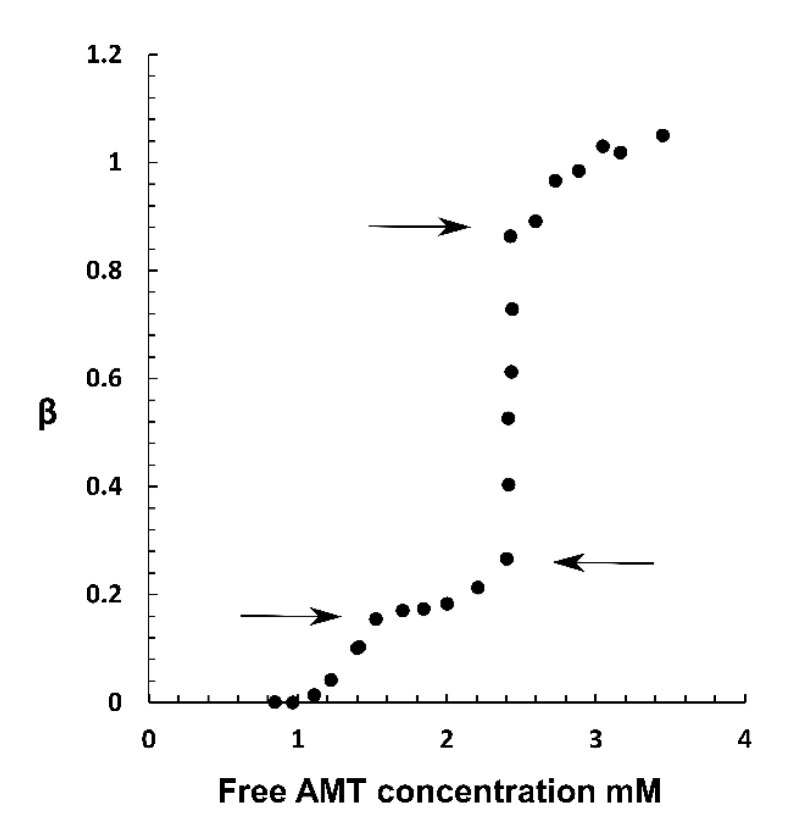
Binding isotherms of AMT in polyacrylate microgels at 155 mM ionic strength. Binding ratio plotted vs. AMT free concentration, which represents the equilibrium concentration of AMT in the liquid. Arrows mark the transitions between different regimes.

**Figure 10:**
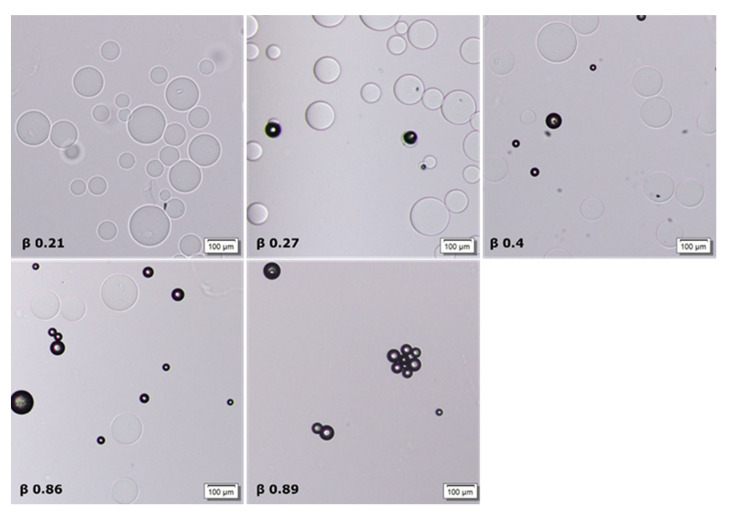
Polyacrylate microgels after equilibration with AMT solution at 155 mM ionic strength. Pictures taken at different binding ratios.

To provide complementary information about the nature of the phase separation at physiological ionic strength, we investigated suspensions of microgels in solutions containing limited amounts of AMT and 155 mM phosphate buffer (pH = 7.4). Figure 9 shows the binding ratio as a function of the free AMT concentration after equilibration for 6 weeks. The free AMT concentration was measured with the µDISS profiler as described in Section 5.5; each point on the binding isotherm derives from a separate experiment. Figure 10 shows microscopy images of the suspensions at different average binding ratios (indicated in the figure). The binding isotherm can be divided into four regimes and the transitions between them are marked with arrows. Going from low to high β, we found that the binding ratio first increased slowly with increasing free concentration. In the second regime, the binding isotherm developed a plateau before it started to slowly increase again. The plateau can be explained by the formation of micelles in the solution. That was confirmed by surface tension measurements showing that the critical micelle concentration (CMC) for AMT was 1.4 mM in 155 mM phosphate buffer solutions, in good agreement with the concentration in equilibrium with the microgels at the beginning of the plateau. Thus the plateau developed because the concentration of free AMT monomers in the solution increased slowly with increasing total concentration above the CMC. However, it continued to increase and at β = 0.27, where the third regime starts, the binding became very cooperative as indicated by the steeply rising binding isotherm at constant AMT concentration of 2.4 mM in the solution. While microscopy images taken from the first two regimes contained only swollen and apparently homogenous microgels, the third region showed coexisting swollen and collapsed microgels, both apparently homogeneous. We observed no semi-swollen microgels or core/shell phase separated microgels. In this regime, the fraction of collapsed microgels increased gradually with increasing binding ratio until all were fully collapsed. The latter point marked the entry into the fourth regime, where the biding ratio continued to increase but in a non-cooperative fashion. At the highest concentration investigated, the binding ratio was larger than unity, meaning that AMT binding was accompanied by binding of negatively charged salt ions. This is in agreement with theoretical model calculations showing that increasing the ionic strength is expected to facilitate binding beyond the point of amphiphile/network charge stoichiometry [47].

To summarize, the bimodal distribution of microgels with fully collapsed microgels coexisting with swollen ones and the steep rise of the binding isotherm in the third regime show that AMT-rich and AMT-lean phases can coexist in equilibrium at the ionic strength of 155 mM.

## 3. Discussion

In a previous paper, we investigated the kinetics of the PA microgel volume change during loading and release of AMT [48]. Micromanipulator studies of single microgels showed that the volume change was accompanied by phase transformation between swollen and collapsed gel states, where, at intermediate stages, the new phase formed a shell surrounding the original phase. The microgels were in contact with a solution of fixed AMT concentration during loading, and with an AMT-free solution during release. Under those conditions the core/shell state is unstable with respect to the position of the phase boundary [40], and so the phase transformation always goes to completion as long as the AMT concentration in the liquid is above and below the critical value for collapse and swelling transitions, respectively. In the present work we have equilibrated single microgels in small liquid volumes containing limited amounts of AMT. We used a specially designed setup (Figure 1) allowing us to capture the systems at intermediate loading levels simply by the depletion of AMT from the solution during loading. This made it possible to determine (i) the relationship between microgel volume and drug load (Figure 2 and Figure 7a), (ii) in what β range the gels contained coexisting phases, and (iii) how the phases were distributed in the microgels. The method is a refinement of an earlier method [31] used to study PA microgels interacting with the cationic surfactant dodecyltrimethylammonium bromide (C_12_TAB). In that method, microgels were placed in aqueous droplets encapsulated by oil to avoid evaporation. The method was not applicable to AMT since the base form of the drug partitioned to the oil phase, and although present at very small fractions in the aqueous phase, substantial amounts of drug escaped from the droplets since the oil phase was in large excess. Other advantages of the present method over the previous are that the risk of emulsifying the oil in the aqueous phase is eliminated, and that the microgel can be inserted into the aqueous solution without being transferred across the oil, thereby also eliminating the risk of covering the microgel with a layer of oil. In the following sections we will discuss the relevance of the results obtained with the new method.

### 3.1. Drug Loading at 10 mM Ionic Strength

The results in Figure 2, Figure 3, Figure 4 and Figure 5 show that dense (collapsed), AMT-rich domains coexisted with swollen, AMT-lean domains in the same microgel particle, and that the phase coexistence remained after long incubation periods. At sufficiently high loadings, both types of domain were so large that there is no doubt that they constituted macroscopic phases. This is in agreement with our previous report of coexisting swollen and collapsed phases in single PA *macrogels* equilibrated in AMT solutions [48], where the biphasic state occurred in approximately the same β-range as for the present microgels. In the same report we showed that PA microgels suspended in AMT solutions were either swollen or collapsed in about the same (average) β-range. In both cases the slope of the binding isotherm was practically infinite in the biphasic range, as expected for 1st order phase transitions, and took place at nearly the same AMT concentration in the solution. However, the phase transition was preceded at lower concentrations by a binding regime of finite cooperativity where AMT formed micelles in the gels. That substantial amounts of micelles can be present in the gels prior to phase transition is borne out by the present results in the lower monophasic β-range, where the volume of the microgels decreased with increasing β (Figure 2) and where rhodamine B accumulated in them (Figure 3).

Equilibrium properties of macrogels are difficult to study since the time to reach equilibrium can be exceedingly long. For microgels however the situation is much improved and the present results provide strong evidence that the biphasic state represents the equilibrium state in the intermediate β-range. Jidheden and Hansson [31] came to the same conclusion after studying PA microgels interacting with C_12_TAB. In our previous paper we noted a striking similarity between AMT and C_12_TAB in the way they interacted with PA macrogels. We can now confirm that this is much the same for microgels. However, the repositioning of the collapsed phase with time from shell to calotte observed here (Figure 5) is a new feature not observed elsewhere for spherical gels.

The micrographs in Figure 5 show that, during accumulation of AMT in the microgel, the collapsed phase first appeared as separate domains in the outermost part of the gels, then developed into a rather intact shell, and then finally “slipped off” to one side to form the calotte. Hence the calotte appeared to be the equilibrium position of the collapsed phase. We only observed calottes for β > 0.5, i.e., when they contained roughly half or more of the network. Moreover, the calotte shape of the phase, with a radius similar to that of the microgel in the fully collapsed homogeneous state (Figure 3 and Figure 5), shows that the state of the network in it was close to that of the “relaxed” network in the fully collapsed homogeneous state. Together the results suggest that the shell-to-calotte rearrangement was driven by elastic deformation energy (“shape memory”). Thus, when a sufficiently large fraction of the network is in the collapsed phase, the shape/deformation of the swollen phase is sacrificed to allow the collapsed phase to assume an optimal shape (lowest elastic energy). In contrast, the calotte is not expected to form at lower β values, where only a minor part of the network is in the collapsed phase. Here, strive to lower the free energy of the swollen phase dominates, and so the collapsed phase forms a shell or other arrangements near the microgel surface where the collapse has the smallest influence on the volume and shape of the swollen phase.

Regular surfactants, such as C_12_TAB, have never been reported to form calottes in PA microgels. Why is that? We believe the dynamics at the microscopic level play a role, since large scale rearrangement requires either that micelles dissolve and reform at other places, and/or high mobility of intact micelles. The fact that the AMT-collapsed phase relocated from shell to calotte on a rather short time scale is an indication of fast dynamics, showing also that the network crosslinks do not prevent the molecules from redistributing inside the gel. This in turn suggests that the patterns of collapsed phase on the microgel surfaces at β = 0.35 (Figure 3) and β = 0.39 (Figure 4) were not “frozen” structures, since AMT is expected to redistribute during the time the pattern was observed. The dynamics are expected to increase with increasing concentration of monomers in local equilibrium with the micelles. Based on the CMCs in water [57,58], one would expect AMT to display faster dynamics than C_12_TAB, since higher free monomer concentration means shorter residence time in micelles. However, both have nearly the same CAC in the present microgel system [48], and one may argue that CAC is more relevant than CMC in this respect. But neither quantity is a measure of the monomer concentration inside the collapsed phase, where it depends also on, e.g., micelle/polyion charge ratio, aggregation number and micelle-micelle interactions. The collapsed phase formed by C_12_TA^+^ is structurally ordered (micellar cubic or hexagonally close packed) while AMT appears to form a disordered micellar phase (unpublished results). The latter is in agreement with a recent small-angle x-ray scattering study [58], showing that the system AMT—H_2_O formed a disordered micellar phase (L_1_), with aggregation number between 34 and 42, even at high AMT concentrations, comparable to those in the collapsed phase in the present microgels. The packing density and phase structure is expected to affect the rate at which the phase relocates in the microgel in response to the elastic network forces acting on it, both by determining the deformability of the phase and by affecting the micelle-monomer equilibrium. It has been suggested elsewhere [43,44,56,59] that differences in the dynamic properties of shells formed by C_12_TAB and C_16_TAB, respectively, in PA macrogels, derive from the cubic phase structure of the former and the hexagonal phase structure of the latter. It can be mentioned that in cylinder shaped PA macrogels, C_12_TAB-collapsed shells have been observed to relocate to a cylindrical part flanking the swollen part, both parts being deformed in the part closest to the interface between them. However, the process took several month to complete.

In the present microgels, the collapsed phase first appeared as discrete domains in the outermost parts (Figure 3 and Figure 5). This is in agreement with the “paradigm” that an insurmountable shear deformation energy barrier prohibits nucleation in the bulk of gels [60]. According to current theory [39,40,61,62], the consequences of that are the following. The network in the collapsed domain is strongly deformed from its preferred isotropic state by the elastic forces transmitted from the swollen “mother” phase. Thus in order for the collapsed phase to nucleate, the system must pay the extra free energy cost of deforming the network. This shifts the CCC to a higher value than that at the so-called Maxwell point, where the collapsed and the swollen phases have the same free energy in their preferred undeformed states, independently of each other (states that are not accessible as long as they are part of the same network). However, as the fraction of the network in the collapsed phase increases, the deformation decreases and by that also the phase coexistence cost. Thus, the AMT concentration in the liquid in equilibrium with the microgel is expected to decrease as β increases above the critical value for nucleation of the collapsed phase. In other words, since it becomes increasingly more favorable for AMT to be in the collapsed phase, not only the AMT molecules added to the system after this point, but also additional ones from the solution become incorporated into the collapsed phase. The interesting question arises as weather AMT molecules also become relocated from the swollen to the collapsed part. A possible indication that this could be the case was the decolorization of the swollen part of rhodamine B-containing microgels, which took place when the calotte was formed (Figure 3 and Figure 5). Recall that substantial amounts of micelles were present in the microgel prior to that point (β ≈ 0.3). One could argue that the swelling of the swollen phase would increase if the concentration of micelles in it decreased. However, the effect could be masked by the compressive forces imposed on it by the network in the collapsed phase. Future confocal Raman spectroscopy investigations of the composition of the swollen phase will hopefully help us answer this question.

The swelling isotherm for PA microgel + AMT (Figure 2) is very similar to the swelling isotherm for PA macrogel + AMT published earlier [48]. This was somewhat surprising at first considering that the macrogels had the form of short cylinders and displayed “regular” core/shell phase separation. However, we noted that the volume of the microgel with β = 0.6, which after ca. 5 h of incubation displayed a core-shell structure, was practically the same after several days when the calotte had formed. It showed that most of the drug had entered the microgel already after a few hours, and that the distribution of the coexisting phases had a relatively small influence on the volume. This may explain the similarity in the swelling profiles of the macrogel and the microgel despite the difference in how the swollen and collapsed phases were distributed.

### 3.2. Phase Separation in Microgels at Physiological Ionic Strength

Earlier work showed [63,64] that polyion-mediated attractions between micelles is largely responsible for the associative phase separation in polyelectrolyte-surfactant mixtures, and that the addition of salt lowers the stability of polyion-surfactant complexes and the tendency for phase separation. At the outset, therefore, it was far from obvious that the electrostatic driving force would be strong enough at physiological ionic strength to cause phase separation in mixtures of AMT and PA microgels. However, the results presented above show that phase separation can take place both within single microgels (Figure 6, Figure 7 and Figure 8) and among the individual microgels in a suspension (Figure 9 and Figure 10). The result is important for understanding the mechanism of drug release from microgels. In our previous paper [48], we studied the release of AMT from PA microgels to a solution of physiological ionic strength under sink conditions. The kinetics of the process was studied indirectly by monitoring the swelling of single microgels at a controlled liquid flow rate in a specially constructed flow pipette. At intermediate stages during the release, a swollen shell was formed with a sharp boundary to the dense core, similar to the microgels at intermediate loading in Figure 6. We successfully modelled the swelling kinetics by assuming that the microgel quickly reestablished osmotic equilibrium with the surroundings at every instance during the release, and that the swelling was rate-controlled by the rate of diffusive transport of AMT molecules through the shell. Thus, the microgel volume was considered to be a function of the amount of AMT remaining in the core rather than a function of time. This allowed us to calculate the radius of the core, the thickness of the shell and the concentration of AMT on each side of the core-shell boundary by means of a thermodynamic equilibrium model. However, we did not account for the stability of the phase boundary as such. The bimodal distribution of swollen and collapsed microgels in the suspension (Figure 10) clearly shows that there is a thermodynamic driving force for phase separation even at an ionic strength of 155 mM in the solution. The results for single microgels in small liquid volumes (Figure 6, Figure 7 and Figure 8) show that swollen and collapsed phases can coexist at equilibrium also in the same microgel. Together the results justify the assumption of a sharp and thermodynamically stabilized phase boundary between the core and the shell in the model calculations. We emphasize however that the collapsed core-swollen shell arrangement may not represent the true equilibrium state of the system. In fact, theoretical calculations show [40] that the elastic forces always favor collapsed shells over collapsed cores (at least at 10 mM salt). If that is true in general, then the collapsed core/swollen shell structure observed at ionic strength 155 mM is not the global free energy minimum. The relocation of the collapsed phase from the core to other positions after long incubation time shown in Figure 8 is an indication of that. However, the core-shell state may still represent a deep local free energy minimum, and thus be stabilized by thermodynamic factors.

In the previous paper the volume was presented as a function of time for microgels releasing AMT to a medium of infinite volume and with the same ionic strength as used here. Here we present in Figure 7b the theoretically calculated relationship between *V/V*_0_ and β underlying the calculations of the release profiles. The kinetic model was based on the assumption that, at each point in time during release, the AMT/PA charge ratio in the core is equal to that of the fully loaded microgel prior to release and that the microgel is in osmotic swelling equilibrium with the surroundings. Comparison of the swelling isotherm in Figure 7b with the experimental one in Figure 7a is therefore a way to validate the performance of the thermodynamic gel model. As can be seen, the theoretical curve lacks the distinct break point between the regimes at high and low binding ratios observed in the experiments, but the gel volume increases faster with decreasing β in the lower binding regime in qualitative agreement with the experiments. According to theory [40], the shell is free to adjust its swelling in the radial direction, but in the lateral direction at the core boundary the network in it is forced to have the same deformation ratio as the core network. At high β, where a major part of the network is in the core, the swelling of the shell is strongly limited by being attached to the collapsed core. Here the shell is considerably less swollen than a free network. However, at low β, where the major part of the network is in the shell, the constraint imposed by the core is smaller, and so the swelling approaches that of a free network more rapidly with decreasing β. We conclude that, although there is room for improvements, the model captures qualitatively the interplay between the core and the shell.

## 4. Conclusions

In this work we have addressed the fundamental question of phase coexistence at equilibrium in polyelectrolyte gels loaded with an amphiphilic drug of opposite charge to the network. The results from microscopy studies show that single PA microgels loaded with limited amounts of AMT contained a dense phase enriched in AMT micelles coexisting with a swollen AMT-lean phase.

After loading with small amounts of AMT (0.26 < β < 0.52) at low ionic strength, the dense phase formed discrete domains near the gel boundary. At higher load (0.52 < β < 0.76), the dense phase initially formed a shell that after further incubation relocated to one side of the microgel in the shape of a calotte. The calotte is a new feature not reported earlier, at high loading presumably representing a configuration of lower elastic energy than a shell. We have demonstrated that the new experimental setup for studies of single microgels in a small liquid volume makes it possible to record swelling isotherms (*V/V*_0_ vs. β) with high precision. The shape of the swelling isotherm reflected the onset of phase separation and displayed the non-linear behavior characteristic of gels with two coexisting, elastically coupled phases.

At physiological ionic strength (155 mM), initially fully loaded microgels developed a swollen shell enclosing the micelle-rich core after partial release of AMT. Although the network in both phases was not fully relaxed, the core-shell separation remained stable long enough for it to be considered as an equilibrium state or a very long-lived metastable state. The swelling isotherm indicated that the swelling of the shell was strongly limited when the majority of the network was in the collapsed core. The non-linear behavior was found to be in qualitative agreement with the results from theoretical model calculations describing the elastic coupling between the phases.

In suspensions of microgels at physiological ionic strength, we observed phase separation between swollen homogeneous and collapsed homogeneous microgels in approximately the same β-range as single microgels contained two phases. We conclude that the driving force for phase separation remains and that swollen and collapsed phases can coexist in the same microgel at physiological ionic strength. Furthermore, we have found evidence that the phase boundary between the swollen shell and collapsed core, previously observed during the dynamic process of drug release, is maintained by thermodynamic interactions rather than as a result of slow dynamics on the molecular level. The results support a release mechanism proposed earlier [36,48] based on successful modelling of the in vitro swelling/release kinetics of drug-eluting beads used in cancer therapy.

## 5. Materials and Methods

### 5.1. Materials

Acrylic acid (anhydrous 99%), *N*,*N*′-methylenebisacrylamide (99%), *N*,*N*,*N*′,*N*′-tetramethylethylenediamine (TEMED) (reagent plus 99%), ammonium persulfate (≥98%), sorbitane monostearate (Span 60^®^), sodium chloride, sodium phosphate monobasic (ReagentPlus^®^ ≥ 99%), sodium phosphate dibasic (ReagentPlus^®^ ≥ 99%), amitriptyline hydrochloride (AMT) (≥98% TLC powder), and rhodamine B were purchased from Sigma-Aldrich (St. Louis, MO, USA). Cyclohexane (≥99.5%) was from Merk (Darmstadt, Germany), sodium hydroxide was from Riedel-de Haën (Sigma-Aldrich, St. Louis, MO, USA) and methanol (HPLC grade) was from Fisher Chemical (Hampton, VA, USA). All chemicals were used as received. Wherever we mentioned water in this work it means Ultrapure (Type 1) water from Synergy^®^ UV Water Purification System (Merck, Darmstadt, Germany).

### 5.2. Microgels Synthesis

Microgels were synthesized by inverse suspension polymerization technique as described in more details elsewhere [48]. The aqueous phase was prepared by mixing 2.6 g of acrylic acid, 109 µL of TEMED, 6.5 g of 80 mM cross-linker *N*,*N*′-methylenebisacrylamide (corresponding to 1.4 mole% of the total amount of monomers), 11 g of 2 M NaOH and water to a final volume of 20 mL. The continuous oil phase was prepared by dissolving 0.09 g of Span 60 in 30 mL of cyclohexane. After mixing for two days under stirring, it was preheated in a two-necked round-bottomed flask to 45 °C under nitrogen atmosphere at 400 rpm stirring. We then mixed 10 mL of the aqueous phase with 364 µL of 0.18 M ammonium persulfate and injected the solution gradually into the oil phase. The temperature was raised to 60 °C under continuous stirring at 1000 rpm. After 30 min we stopped the reaction by adding methanol. After that, methanol was removed from the mixture and the concentrated microgel suspension was dried at 60 °C by using Carbolite Furnaces airflow. Finally, the dried microgels were suspended in water and rinsed four times with water. After confirming that the pH > 9 in the microgels suspension, the microgel suspension was stored in a refrigerator.

### 5.3. Swelling Isotherm in 10 mM Ionic Strength

A specially constructed microscopy cell was used to equilibrate each microgel in a small volume, typically 3–15 µL, of AMT solution as shown in Figure 1. We used a Zeiss Axio Vert.A1 inverted microscope with AxioCam MRc camera and Zeiss 10×/0.25 lens (Jena, Germany) equipped with a Narishige M-152 and IM-11-2 (Tokyo, Japan). Narishige micropipette puller PN-31, micropipette grinder EG-400, and micro-forge MF-900 (Tokyo, Japan) were used to prepare the gel holder. The cell consists of a glass tube with a diameter of 1.5 mm inserted in a petri dish. The petri dish was positioned over the inverted lens in the microscope.

One single microgel was picked by a micro holder and positioned in the AMT solution inside the tube. To prevent evaporation, the tube was then sealed by oil drops with no contact with the solution. The petri dish was filled with water to prevent optical reflections. We followed the progression of the system by taking pictures of the microgel during the incubation period, which typically lasted for ca. 22 h, but in some cases longer (two to five days). In order to obtain different binding ratios, we combined microgels of different size with solutions of different AMT concentration and volume. The binding ratio was calculated by using the equation:

(2) $$\beta =\frac{C_{AMT}^{total}-C_{AMT}^{free}}{C_{PA}^{gel}}$$

where $C_{AMT}^{total}$ is the concentration of AMT in the solution before inserting the microgel, $C_{AMT}^{free}$ is the AMT concentration in the solution in equilibrium with the microgel, and $C_{PA}^{gel}$ is the concentration of acrylate segments in the system. $C_{AMT}^{total}$ was corrected by subtracting the amount of AMT absorbed on the glass tube. The latter was determined in the following way. First, we used a µDISS profiler from Pion Inc. (East Sussex, UK) to measure the concentration of AMT in a test solution, before adding it to the glass tube. We then filled the glass tube with the solution. After that, we collected the solution in a µDISS profiler tube and measured the concentration. The difference in the concentration was used to estimate the amount bound to the glass tube.

Depending on the shape of the gel after equilibration we assumed either spherical, ellipsoid, or spherical cap geometry to calculate the volume of the gel. After that, the volume *V* of the gel after equilibration in AMT solution was divided by the volume *V*_0_ of the gel in 10 mM phosphate buffer to get the volume ratio *V*/*V*_0_. Fluorescence microscopy was used to study the phase separation and the distribution of AMT inside the gel. Rhodamine B was used as a fluorescent probe with a ratio of 1 molecule for every 200 AMT molecules. Filter set 43 was used with excitation/emission band-pass 545/605 nm.

### 5.4. Swelling Isotherm at 155 mM Ionic Strength

We used the same principle as mentioned above. A single microgel was positioned in 0.7 mM AMT solution of 10 mM ionic strength overnight to load the drug with AMT. Then the loaded gel was positioned in a small volume of the desired AMT solution in 155 mM ionic strength. By controlling the concentration and the volume of the AMT solution, we could control the amount released from the gel to get the desired binding ratio. The gel was equilibrated for 5 h and images were captured by Zeiss Axio Vert.A1 light microscope. After equilibration, the gel was moved to 250 µL of 155 mM phosphate buffer to release the remaining AMT from the gel to the buffer solution. Then we measured the concentration of AMT in the buffer solution, which corresponded to the AMT content inside the gel, by using µDISS profiler. The measured concentration was corrected by measuring the amount lost due to moving the solution from the glass tube to the µDISS tube. The µDISS fiber optic probe length was 20 mm and the range of the wavelength was 255–265 nm. The swelling isotherm was determined by plotting the volume ratio *V*/*V*_0_ as a function of the binding ratio, where *V*_0_ is the volume of the gel after being fully loaded with AMT, and *V* is the actual volume after equilibration.

The main difference in the experimental set-up from 10 mM experiment is that the gel holder’s side was sealed with water instead of an oil drop. After equilibration, the water drop was wiped off before removing the gel from the tube. In this way, we were able to withdraw the gel from the tube without any contact with an external solution.

### 5.5. Binding Isotherm in Microgel Suspension at 155 mM Ionic Strength

Freeze dried microgels were dispersed in 50 mL of 155 mM phosphate buffer solution. Then AMT was added to the dispersion and equilibrated for 6 weeks. Different intermediate loading levels of microgels were reached by choosing different concentrations of AMT. After equilibration, the dispersion was filtered by 40 µm filter to separate the microgels from the solution. Then the concentration of the free AMT in the solution was measured by using a µDISS profiler. The fiber optic probe length was 5 mm and the range of the wavelength was 290–296 nm. The amount bound AMT was calculated from the difference in the concentrations between the total AMT and the free AMT. Then the binding ratio was calculated from the amount bound AMT and the mass of the freeze dried microgels.

### 5.6. The Concentration of Polyacrylate Segments in Microgels

PA microgels were freeze-dried and weighed by Mettler Toledo MT5 microbalance. The remaining moisture content was measured by Differential scanning calorimeter (DSC Q 2000, TA instruments). The weight was corrected for the remaining moisture content. Then we dispersed the microgels in water in a small sealed cell and positioned it under Olympus Bx-51 light microscope. The radius of all microgels in the cell was measured and the average concentration of the network charges inside microgels was obtained by dividing the weight of polyacrylate microgels with the total volume of the dispersed microgels in water.

### 5.7. Determination of CMC

A sigma 703D force tensiometer equipped with Wilhelmy plate was used to measure the surface tension of different concentrations of AMT solutions in a 155 mM phosphate buffer. The measurements were performed at room temperature.
